# Haemophagocytic Lymphohistiocytosis Associated With Anaplastic Large-Cell Lymphoma in a Young Woman

**DOI:** 10.7759/cureus.35130

**Published:** 2023-02-18

**Authors:** Fernando Nogueira, Isabel C Brito, Catarina V Pereira, José C Marques, Ester Ferreira, Ana Carneiro

**Affiliations:** 1 Internal Medicine, Centro Hospitalar Universitário de São João, Porto, PRT; 2 Clinical Haematology, Centro Hospitalar Universitário de São João, Porto, PRT

**Keywords:** fever, pancytopenia, peripheral t-cell lymphoma, haemophagocytic lymphohistiocytosis, anaplastic large-cell lymphoma

## Abstract

Haemophagocytic lymphohistiocytosis is a syndrome of excessive immunological activation that can be triggered by various diseases, including haematological cancers. We report a case of a 25-year-old woman presenting with constitutional symptoms and a painful thoracic mass of four months duration. Laboratory exams showed pancytopenia, hypertriglyceridemia and extremely high serum ferritin levels. A whole-body computed tomography (CT) scan revealed splenomegaly and highlighted the mass on the deep tissues of the left breast; the biopsy was compatible with anaplastic large-cell lymphoma. Additionally, a bone marrow biopsy revealed haemophagocytosis, fulfilling the criteria for associated haemophagocytic lymphohistiocytosis. The patient was quickly sent for chemotherapy followed by autologous haematopoietic cell transplantation. She achieved a complete metabolic response and has been in clinical remission after nearly four years of follow-up. We emphasise the benefit of a timely diagnosis and intervention which were the keys to success in this case.

## Introduction

Haemophagocytic lymphohistiocytosis (HLH) is a rare syndrome of disproportionate immune activation associated with hyperinflammation and tissue destruction [1]. HLH is primarily a paediatric syndrome, although it can be observed in patients of all ages [2]. It can occur as familial or sporadic forms and be triggered by a variety of conditions, including infections (mostly viral), malignancies (typically lymphoid cancers and leukaemias), rheumatological diseases (such as systemic juvenile idiopathic arthritis) or inherited and acquired immunodeficiencies [3]. Despite the increasing number of cases reported in the literature during the last decade, it is still a rare entity and its incidence in adults is unknown [3].

Herein we describe the case of a young woman in whom the diagnosis of HLH associated with a de novo anaplastic large-cell lymphoma (ALCL) was made.

This case report was previously presented as a poster at the Portuguese 25^th^ National Internal Medicine Congress (Congresso Nacional de Medicina Interna) in May 2019 and at the 19^th^ European Congress of Internal Medicine in March 2021.

## Case presentation

This is a case report of a 25-year-old Caucasian female with unipolar major depression medicated with sertraline 50 mg QD and alprazolam 0.25 mg PRN. Her personal and familiar medical history revealed no other remarkable findings.

She presented to the emergency department with a four-month course of worsening fever, asthenia, anorexia, and unintentional weight loss (20% of total body weight in four months), as well as a painful mass in the anterior left hemithorax. The epidemiological background was unremarkable. Previously, she was submitted to several laboratory and imaging exams, as well as a core biopsy of the mass, all of which were inconclusive. Furthermore, the patient did not improve with a course of non-steroidal anti-inflammatory drugs or empiric antibiotics (amoxicillin-clavulanate). On physical examination, we highlight high-grade fever (39.2 °C), reactive tachycardia, low body mass index (16.2 kg/m^2^), pallor and a painful mass on the left upper quadrant of the left breast, without palpable lymphadenopathies, hepato- or splenomegaly.

Positive laboratory results included pancytopenia (normocytic normochromic anaemia, leukopenia, severe neutropenia, and moderate thrombocytopenia) as well as hypoalbuminemia, hepatic cytolysis without cholestasis, elevated lactic dehydrogenase (LDH), discretely elevated C-reactive protein, hyperferritinemia, fasting hypertriglyceridemia and slight hypofibrinogenemia. Urine and blood gas analyses were unremarkable. Detailed laboratory results at admission and during follow-up are presented in Table 1. Chest radiograph and abdominal ultrasound showed no abnormalities, while a breast ultrasound suggested mastitis. Blood and urine cultures were drawn, and intravenous piperacillin/tazobactam 4.5 g q6h was started empirically. She was then hospitalized for further investigation and treatment.

**Table 1:** Basic laboratory tests performed during diagnosis, treatment, and follow-up ALT – alanine aminotransferase; AP – alkaline phosphatase; AST – aspartate aminotransferase; CHOEP – chemotherapy regimen with cyclophosphamide, doxorubicin, vincristine, etoposide and prednisone; CPR – C-reactive protein; GGT – gamma-glutamyl transpeptidase; HCT – haematopoietic cell transplantation; LDH – lactate dehydrogenase.

Parameter	Unit	Reference	On admission	After 1 cycle of CHOEP	After 6 cycles of CHOEP	100 days after HCT	1 year after HCT	4 years after diagnosis
Haemoglobin	g/dL	12.0-16.0	8.2	10.6	11.0	11.7	12.8	12.4
Leucocytes	x10^9^/L	4.0-11.0	0.9	1.19	1.8	4.1	4.9	3.7
Neutrophils	x10^9^/L	2.2-7.7	0.3	–	0.7	2.7	2.9	1.5
Lymphocytes	x10^9^/L	0.9-4.0	0.5	–	0.7	1.1	1.4	1.7
Platelets	x10^9^/L	150-400	68	61	206	182	209	170
Total protein	g/L	64.0-83.0	65.1	66.5	67.2	68.9	71.2	68.1
Albumin	g/L	38.0-51.0	31.9	35.6	38.8	42.8	41.3	43.0
AST	IU/L	10-31	201	27	20	20	16	21
ALT	IU/L	10-31	114	44	12	13	12	16
GGT	IU/L	7-32	75	93	10	14	11	11
AP	IU/L	30-120	82	84	73	89	92	60
Bilirubin	mg/dL	<1.20	0.74	0.56	0.41	0.42	0.39	0.41
LDH	IU/L	135-225	1249	365	156	168	123	164
Glucose	mg/dL	75-110	100	87	107	83	88	95
Urea	mg/dL	10-50	16	17	29	29	28	37
Creatinine	mg/dL	0.51-0.95	0.36	0.34	0.49	0.62	0.69	0.67
Uric acid	mg/dL	2.3-6.1	<1.5	<1.5	3.3	3.6	3.1	3.8
Sodium	mEq/L	135-147	136	138	146	140	141	138
Potassium	mEq/L	3.5-5.1	3.9	4.0	4.0	4.1	3.6	4.6
Chloride	mEq/L	101-109	105	105	110	105	106	103
CPR	mg/L	<3.0	3.7	3.7	<0.2	8.2	9.7	0.7
Ferritin	ng/mL	10-120	5567	–	77.7	268.1	–	–
Triglycerides	mg/dL	10-31	322	–	–	–	–	–
Fibrinogen	mg/dL	200-400	196	–	435	309	314	–

**Figure 1:**
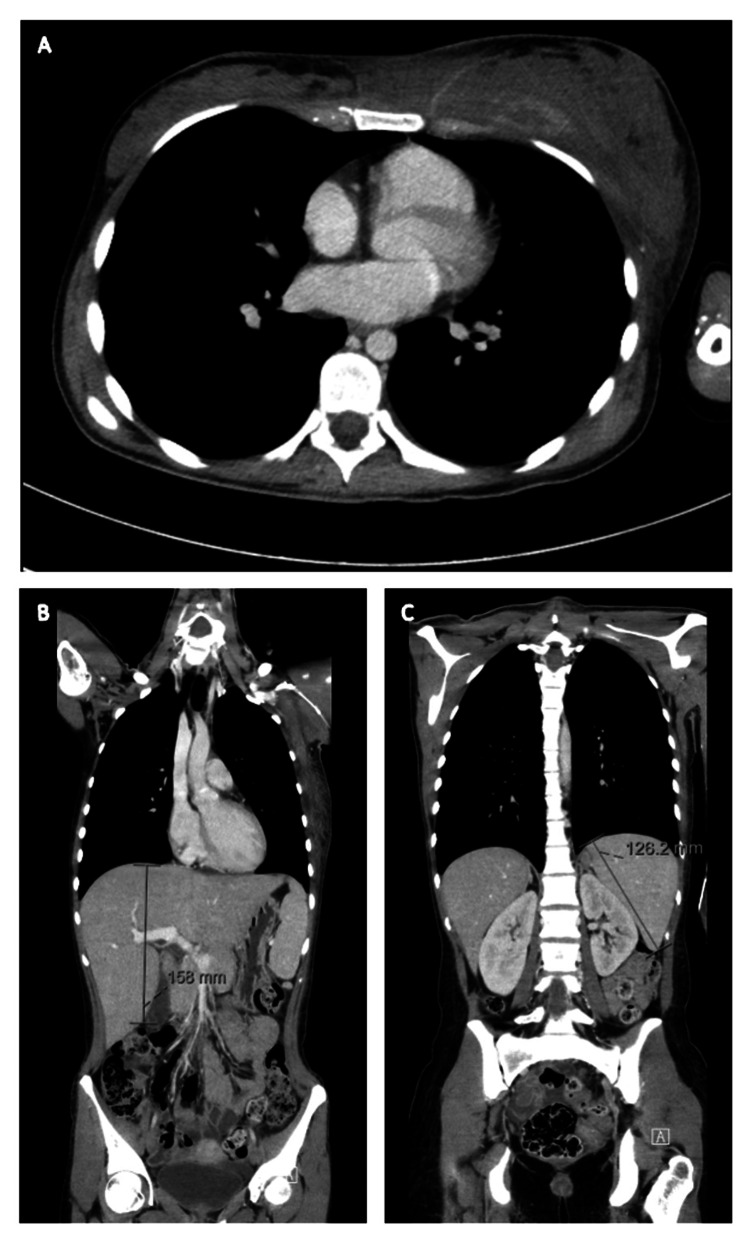
Whole-body computed tomography scan showing a mass with 8 x 3 cm in the deep tissues of the left breast with extension to the pectoralis major muscle (panel A), as well as slight hepatomegaly (panel B) and splenomegaly (panel C).

**Figure 2:**
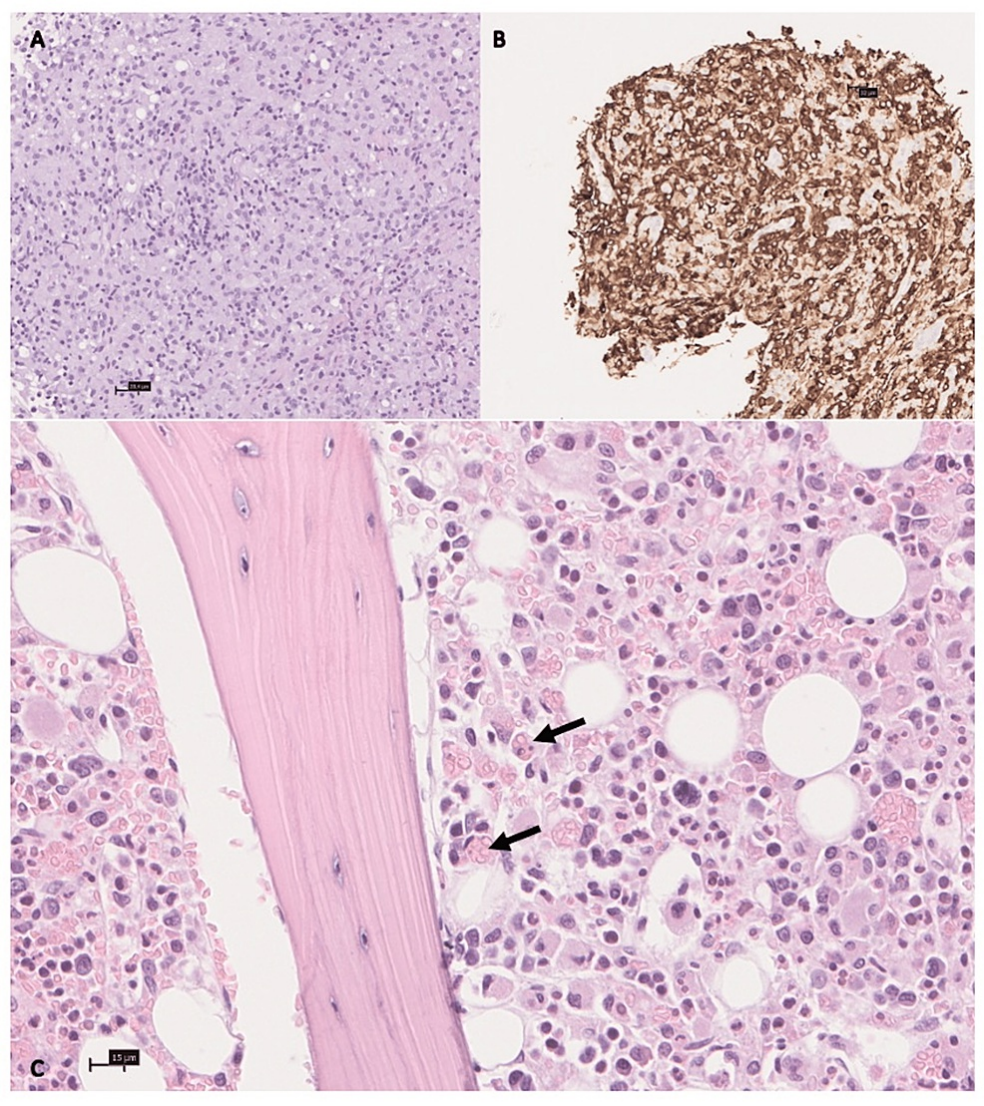
Histopathological exam of the mass showing large neoplastic cells in a background of small lymphocytes and histiocytes (panel A); the neoplastic cells express CD30 (panel B), compatible with large-cell anaplastic lymphoma. Bone marrow biopsy revealed numerous hemophagocytic histiocytes, represented by black arrows (panel C).

A whole-body computed tomography (CT) scan showed a mass of 8 x 3 cm in the deep tissues of the left breast, with extension to the pectoralis major muscle, together with slight hepatomegaly (16 cm) and splenomegaly (13 cm) (Figure 1). Blood and urine cultures were negative as well as serologies for multiple pathogens. An immunological panel and peripheral blood immunophenotyping were also performed and were normal. The patient maintained daily high-grade fever despite antibiotic therapy. A core-biopsy of the mass was repeated which later revealed muscular involvement by anaplastic lymphoma kinase (ALK)-positive ALCL (Figures 2A-2B). Bone marrow biopsy was also performed, showing extensive haemophagocytosis, without signs of lymphoma infiltration (Figure 2C). Additionally, a whole-body positron emission tomography (PET) scan with fluorodeoxyglucose (FDG) showed extensive FDG-avidity on the extranodal mass located inside the left pectoralis major muscle and left adrenal gland, as well as diffuse avidity in the spleen and skeleton.

Given the established diagnosis of ALCL, induction therapy with a CHOEP regimen (cyclophosphamide, doxorubicin, vincristine, etoposide and prednisolone) was initiated, with consequent fever resolution and a slight improvement in cytopenias. The patient was discharged after the first cycle and completed six CHOEP cycles, with peripheral blood precursor cells mobilization at the last cycle. By the end of the treatment, PET-FDG scan was repeated showing a complete metabolic response. Consolidation therapy with autologous haematopoietic cell transplantation (HCT) with BEAM conditioning regimen (carmustine, etoposide, cytarabine and melphalan) was followed. The patient has now almost four years of follow-up without any evidence of relapse.

## Discussion

The reported case is about a young woman with HLH associated with a de novo diagnosis of ALCL stage IV-B.

ALCL is a lymphoid neoplasm of T cell or null cell origin, being one of the most common forms of peripheral T cell lymphoma, accounting for approximately 2% of adult non-Hodgkin lymphoma [4]. Its incidence is higher in children and young adults, with male predominance [5]. On diagnosis, most patients have widespread disease and B symptoms, resembling the presented case. A tissue biopsy is essential for the diagnosis, as it shows a typical histological pattern, as well as CD30 expression on immunohistochemical analysis [6]. This type of non-Hodgkin lymphoma has a particularly aggressive course. The presence of an ALK rearrangement is evaluated on diagnosis since it is associated with better treatment response and has a prognostic impact [5]. In addition to ALK expression, the International Prognostic Index (IPI) is also an important tool for prognosis assessment [7]. In this case, the patient had an IPI score of 3 (based on elevated LDH, stage IV of Ann Arbor and more than one extranodal site) associated with a five-year overall survival of 68% in patients with ALK-positive ALCL. The association of ALCL with HLH has a particularly negative effect on prognosis [8].

HLH can occur as the initial presentation of an underlying disorder, as in this case, or later in the course of the disease. It usually presents as a febrile condition associated with multiple organ involvement but its signs and symptoms are non-specific, making the diagnosis of HLH a challenge. The mostly used diagnostic criteria were introduced in the HLH-2004 protocol; it is only validated for the paediatric population and is listed in Table 2 [9]. Despite that, they are widely applied to adults, since there is no validated set of diagnostic criteria for this population. In the presented case, even though we did not evaluate natural killer cell activity or soluble CD25, our patient still fulfilled sufficient criteria for the diagnosis of HLH (6 out of 8). Among these criteria, she presented with haemophagocytosis, however, it is important to emphasize that its presence is neither necessary nor pathognomonic for the diagnosis. On the other hand, a very high serum ferritin level is common and is helpful in suggesting HLH [10]. Another useful way of accessing the probability of HLH is by calculating the “HScore”, a tool that incorporates points for immunosuppression, fever, organomegalies, triglycerides, ferritin, alanine aminotransferase and fibrinogen [11]. Applying this tool to our patient, a score of 268 points was achieved, giving her a probability of HLH higher than 99%.

**Table 2:** Diagnostic criteria for haemophagocytic lymphohistiocytosis (HLH) used in the HLH-2004 trial. [9] CD25 — cluster of differentiation 25; HLH — haemophagocytic lymphohistiocytosis; IL-2 — interleukin 2; NK — natural killer.

The diagnosis of HLH can be established if one of either A or B below is fulfilled
A: A molecular diagnosis consistent with HLH
B: Five out of the eight criteria below:
Fever ≥ 38.5 ^o^C
Splenomegaly
Cytopenias (affecting at least 2 of 3 lineages of the peripheral blood):
Haemoglobin < 9 g/dL
Platelets < 100 x 10^9^/L
Neutrophils < 1.0 x 10^9^/L
Hypertriglyceridemia and/or hypofibrinogenemia:
Fasting triglycerides ≥ 265 mg/dL
Fibrinogen ≤ 150 mg/dL
Haemophagocytosis in bone marrow or spleen or lymph nodes
Low or absent NK-cell activity
Ferritin ≥ 500 ng/mL
Soluble CD25 (i.e., soluble IL-2 receptor) ≥ 2,400 U/mL

The natural history of untreated HLH is almost uniformly fatal [12]. A high degree of suspicion is needed, and it prompts recognition and diagnosis of the underlying cause or trigger, as these are essential to enable immediate and appropriate treatment, and thus change the natural course of the syndrome. Treatment of HLH depends mainly on the clinical status. For acutely ill patients, treatment is based on the HLH-94 protocol (i.e., etoposide and dexamethasone) [13]. For stable patients without organ dysfunctions, treatment is directed towards the suspected underlying condition or trigger, which is occasionally sufficient to halt the immune dysregulation [3]. This includes antimicrobials for infections, glucocorticoids for rheumatological disorders or antineoplastics for cancers.

In the case of ALCL, most of the cases express CD30, which can be targeted by the brentuximab vedotin (BV). Hence, the preferred induction chemotherapy regimen for ALCL with 10% or more of cells expressing CD30 is with six cycles of BV plus CHP (cyclophosphamide, doxorubicin, and prednisone) [14]. If the expression of CD30 is inferior to 10% in younger (60 years or younger) and medically fit patients, a CHOEP regimen is favoured [15]. Response assessment should be documented including a PET-FDG scan six to eight weeks after completion of induction chemotherapy [16]. Consolidation treatment in ALCL can include salvage chemotherapy and/or autologous HCT. At the time of this case’s presentation, brentuximab was not yet approved nor available for ALCL treatment. So, induction therapy with a CHOEP regimen was the preferred approach and autologous HCT in the first remission was the chosen consolidation therapy.

Despite the worse prognosis predicted ad initium, the patient achieved a complete metabolic response. After almost four years of follow-up, the patient maintains in remission.

## Conclusions

Despite the increase in the number of HLH cases described in the literature over the past decade, this is still a rare, misunderstood and commonly misdiagnosed entity. It is important to recognize the classical signs, symptoms and laboratory findings, to achieve a diagnosis and start therapy promptly. Notwithstanding its bad prognosis, timely identification and management of associated conditions favours the possibility of improved outcomes, as depicted by the presented case.
